# Production of bacterial cellulose from glycerol: the current state and perspectives

**DOI:** 10.1186/s40643-021-00468-1

**Published:** 2021-11-29

**Authors:** Peteris Zikmanis, Sergejs Kolesovs, Maija Ruklisha, Pavels Semjonovs

**Affiliations:** Laboratory of Industrial Microbiology and Food Biotechnology, Institute of Biology, University of Latvia, 4, Ojara Vaciesa Str., Riga, LV-1004 Latvia

**Keywords:** Bacterial cellulose, Acetic acid bacteria, *Komagataeibacter* spp., Glycerol, Sugar alcohols

## Abstract

Current research in industrial microbiology and biotechnology focuses on the production of biodegradable microbial polymers as an environmentally friendly alternative to the still dominant fossil hydrocarbon-based plastics. Bacterial cellulose (BC) is important among microbial polymers due to its valuable properties and broad applications in variety of fields from medical to industrial technologies. However, the increase in BC production and its wider deployment is still limited by high costs of traditionally used raw materials. It is therefore necessary to focus on less expensive inputs, such as agricultural and industrial by-products or waste including the more extended use of glycerol. It is the environmentally harmful by-product of biofuel production and reducing it will also reduce the risk of environmental pollution. The experimental data obtained so far confirm that glycerol can be used as the renewable carbon source to produce BC through more efficient and environmentally friendly bioprocesses. This review summarizes current knowledge on the use of glycerol for the production of commercially prospective BC, including information on producer cultures, fermentation modes and methods used, nutrient medium composition, cultivation conditions, and bioprocess productivity. Data on the use of some related sugar alcohols, such as mannitol, arabitol, xylitol, for the microbial synthesis of cellulose are also considered, as well as the main methods and applications of glycerol pre-treatment briefly described.

## Introduction

With the current development of microbial biotechnology, more attention is focused on the expanded use of renewable resources, which is in line with the general concept of a circular economy and means that the waste products of one industry should serve as the raw material for another (Ravindran and Jaiswal 2016; Schilling and Weiss 2021). Such an approach is of particular importance in regard to inexpensive carbon sources for commercially relevant strains of microbes-producers, since the costs of nutrient media largely determine the overall economic efficiency of bioprocesses (Gahlawat and Srivastava 2017; Sperotto et al.^2021^). For this purpose, numerous by-products, residues and waste of agricultural, food or biofuel industries are proposed and actually applied (Arancon et al. 2013; Sadh et al. 2018; Tsang et al. 2019; Sperotto et al. 2021), which, in addition to the real economic benefits, also create a very positive impact on the environment. Among them, glycerol, the simplest 3-C polyol, as a sufficiently widely available and relatively cheap compound, is already of significant value and is quite promising in this regard. This is largely due to the fact that the production of biodiesel as well as other products (soap, fatty acid, fatty ester industries) whose technologies involve the triglyceride trans-esterification reaction produces significant amounts of glycerol as a by-product (Kumar et al. 2019). It is also important that many microbial cultures are able to utilize it efficiently for the growth and biosynthesis of high added value compounds (Kenar 2007; da Silva et al. 2009; Wendisch et al. 2011; Trindade et al. 2015). These reasons, as well as the ability of a number of producing strains for the use of crude glycerol help to reduce the cost of both the nutrient composition and the end product of microbial synthesis. These products, the biosynthesis of which is based on glycerol as the sole carbon source, represent a fairly wide and versatile set of commercially important compounds such as 1,3-propanediol, ethanol, D-lactic, citric, succinic, propionic acids, glycolipid-type bio-surfactants, carotenoids, amino acids and others (Wendisch et al. 2011; Posada et al. 2012; Yang et al. 2012). Thus, among the glycerol-based products of biosynthesis, there are mainly monomers with a relatively simple structure, but the possibilities of obtaining more complex compounds with a high technological potential are reported much less frequently. In particular, it should be noted that the use of renewable sources is especially important for the biosynthesis of microbial polymers, including extracellular polysaccharides, since reducing overall costs is the main precondition for expanding their production and commercial use (Gahlawat and Srivastava 2017; Sperotto et al. 2021). Microbial polymers are an established environmentally friendly alternative to still dominant fossil-based plastics, possess an extensive biotechnological potential and are already widely used in a variety of fields ranging from medicine to technology (Rehm 2010; Narancic and O’Connor 2019; Shanmugam and Abirami 2019; Zikmanis et al. 2020a, b). However, the scope of research, as well as the number of microbial polymers synthesized on glycerol, is more limited than for monomeric compounds, and in addition they are rather unevenly distributed. Thus, the most comprehensive in this regard are studies on the microbial synthesis of polyhydroxyalkanoates (PHA), and in particular polyhydroxybutyrate (PHB) which are the commercially important biodegradable polymers with a wide range of applications. The key issues of conducting and technological implementation for the relevant bioprocesses have been summarized and assessed in a number of reviews, including those in recent years (Licciardello 2017; Adeleye et al. 2020; El-Malek et al. 2020; Sen and Baidurah 2021; Sirohi et al. 2021). Unlike the formation of microbial exopolysaccharides, using glycerol as the sole carbon source still remains less documented. So there are only sporadic reports on the biosynthesis of gellan (Raghunandan et al. 2018) xanthan (Trindade et al. 2015) and some partially identified heteropolysaccharides (Freitas et al. 2011). Bacterial cellulose (BC) is the only commercially important exopolysaccharide (Gorgieva and Trček 2019), whose microbial synthesis on glycerol has been performed for a relatively long time (Masaoka et al. 1993), and therefore a certain amount of data have been accumulated. However, in specialized reviews on bacterial cellulose, such the employment of glycerol is only noted (Cacicedo et al. 2016; Jang et al. 2017; Reiniati et al. 2017; Ul-Islam et al. 2020) or not mentioned at all (Gullo et al. 2018; Salihu et al. 2019; Zhong 2020), and the available data remain not summarized as well as not evaluated in comparison. However, there is a certain desire for this, albeit to a limited extent (Adnan 2015; Hussain et al. 2019; Mangayil et al. 2021). This review summarizes the current knowledge on the use of glycerol to obtain bacterial cellulose, including information about producer cultures, composition of culture media, cultivation conditions and productivity of bioprocesses. The synthesis of BC from some other sugar alcohols (mannitol, arabitol, xylitol) is briefly overviewed. The basic data on the relevant pathways of biosynthesis and their regulation, properties and applications of BC also are considered.

## Basic features and applications of bacterial cellulose

BC makes up a significant part among other bacterial exopolysaccharides and represents a polymer of D-glucose, that is, a homopolysaccharide formed mainly by β-(1,4) glycosidic bonds from precursors (UDP-D-glucose) (Gorgieva and Trček 2019) through the membrane-incorporated glycosyltransferase (cellulose synthase BcsA; EC 2.4.1.29) (Azuma et al. 2009; Lu et al. 2020). Due to their unique three-dimensional network structure, BC has a combination of exclusive properties such as high crystallinity and degree of polymerization, large surface area, high elasticity, tensile strength and water retention (Wang et al. 2018; Raghavendran et al. 2020; Cazon and Vazquez 2021). BC represents a high-quality and biocompatible material, since it does not contain lignin, hemicellulose, pectin and other biogenic substances, is not cytotoxic and genotoxic. This is particularly important for BC biomedical applications (Ul-Islam et al. 2015; de Oliveira Barud et al. 2016; Moniri et al. 2017; Andriani et al. 2020) in high value-added products (functionalized wound dressings, tissue, heart valves and blood vessels replacement materials, vascular grafts, dental implants, pharmaceutical transfer agents, biosensor elements, etc.). It also appeared to be very promising for use in cosmetics and personal care products (Pacheco et al. 2018; Morais et al. 2019; Bianchet et al. 2020). BC has a multifaceted technological potential (Andriani et al. 2020) and is already used in various industries and production areas, such as textile and paper (Chawla et al. 2009; Pathak and Prasad 2014), mining and refinery (Chawla et al. 2009; Jozala et al. 2016), electronics, energy and communication (Chawla et al. 2009; Baptista et al. 2013; Cacicedo et al. 2016; Li et al. 2019) and waste treatment (Brandes et al. 2018), and especially in food production. BC is used (Shi et al. 2014; Azeredo et al. 2019; Ul-Islam et al. 2020) as an additional thickening, suspending or stabilizing agent to affect the food properties in the desired way or used directly for food (traditional dessert, vegetarian meat, food/beverage additives, etc.) as an ingredient in fiber-enriched low-calorie and low-cholesterol diets. It is also very important that the effectiveness of such a variety of applications can be enhanced by targeted modification of the bacterial cellulose properties. This, in turn, can be achieved through a number of different approaches and techniques. Thus, the high hydroxyl content facilitates the formation of multiple BC derivatives using appropriate chemical (esterification, etherification, amination, oxidation, crosslinking, etc.) reactions (Cook 2013; Reiniati et al. 2017; Gorgieva and Trček 2019) or physical (ultrasonic, rotary magnetic field, UV or γ-irradiation) treatments (Huang et al. 2014; Campano et al. 2016; Blanco Parte et al. 2020). Extensive possibilities also arise when the features of BC are modified and improved by using composite materials of two or more ingredients, which leads to their synergistic interaction and enhancement of the resulting properties (Rosa and Lenz 2013). The need for composites is determined by the fact that certain properties of BC are limited for certain application and must be fine-tuned to match the necessary requirements, particularly for biomedical use (Fu et al. 2013; Feldman 2015). Composites consisting of BC and other compounds including microbial EPS can be obtained by different processing techniques over a wide range of homogenization conditions up to high pressures and elevated temperatures. However, milder conditions are preferable to avoid any possible destruction (Siro and Plackett 2010; Rosa and Lenz 2013). A relative simple and quite efficient method is an augmentation of BC by immersion in solutions of various, preferably water-soluble, compounds including microbial EPS. The solutions after mixing with the suspension of cellulosic particles and evaporation or freeze-drying yield composites with a wide range of modified properties (Yasuda et al. 2005; Millon and Wan 2006; Kim et al. 2011). In this case, molecules of soluble compound not only cover the BC fibrils surface, but also penetrate into the fiber network and bind to BC with hydrogen bonds (Fu et al. 2013). As a result, ex situ post-modifications of the BC to obtain corresponding composites (Klemm et al. 2006) are carried out. Other very promising approach involves the alteration of BC morphology during its biogenesis. Through this approach bacterial cellulose is modified in situ, i.e., during its biosynthesis, due to the competitive adsorption of a compatible host polymer from the growth medium. Therefore, subsequent to BC polymerization, the compounds added to the medium associate and co-crystallize with BC, creating the intimately blended composite materials which can offer a wide range of morphological, compositional and functional properties for specific applications (Brown and Laborie 2007; Rosa and Lenz 2013). Importantly the addition of water-soluble polysaccharides to the cultivation medium itself can appear as the BC synthesis stimulating factor (Chao et al. 2001; Ishida et al. 2003). Alike for other microbial polysaccharides, the structure and properties of BC depend on the cultivation conditions and particular characteristics of the producer culture. Although the ability to synthesize BC is relatively widespread (the species of *Agrobacterium*, *Aerobacter*, *Achromobacter*, *Azotobacter*, *Rhizobium*, *Sarcina* and others), the most important and industrially employed producer cultures are AAB from the genus *Komagataeibacter* (formerly known as *Gluconacetobacter spp*. and *Acetobacter spp*.), mainly the *K. xylinus* and *K. rheticus* strains (Ummartyotin and Sain 2016; Reiniati et al. 2017; Semjonovs et al. 2017a; Gorgieva and Trček 2019; Raghavendran et al. 2020). In addition, producers from different genera and species form BC with elements (pellicles, fibrils, ribbons) of different shapes and sizes (Ummartyotin and Sain 2016). The BC biosynthesis is a rather complex process, which depends on the full composition of the cultivation medium (carbon, nitrogen, micronutrient sources and concentrations), as well as on the culture (temperature, pH, agitation, aeration, growth phase) and operational (bioreactor type and mode of operation) (Mohite et al. 2017; Ul-Islam et al. 2017; Wang et al. 2018; Barcelos et al. 2019; Raghavendran et al. 2020; Tiwari et al. 2020). In general, the bioprocess is also complicated by the strong evidence that the yield and physico-chemical characteristics of produced BC not only depend on the aforementioned factors, but also the magnitude and direction of their complex effects are highly strain-dependent. This, in turn, creates the need to find and define the most appropriate cultivation conditions for each individual producer strain (Olivas and Barbos-Canovas 2005; Nwodo et al. 2012; Devi and Alamu 2013; Semjonovs et al. 2017b), even if the same microbial species synthesizes the same EPS. However, a number of generalized features and conditions promoting

## Sugar alcohols as nutrients for acetic acid bacteria

AAB are characterized by their ability to oxidize not only carbohydrates and alcohols, including glycerol, but also sugar alcohols (polyhydric alcohols or polyols) into organic acids, aldehydes or ketones to gain energy in a process termed “oxidative fermentation”, with some of the genera forming BC (Taban and Saichana 2017; La China et al. 2018; Lynch et al. 2019). The most important of these are the 6-carbon (6-C) mannitol and the 5-C stereoisomers arabitol, xylitol and ribitol. In this respect, mannitol has long been used relatively more frequently and successfully (Minor et al. 1954), when the formation of C14-labeled BC from D-mannitol-1-C14 by *A. xylinum sp.* was confirmed. The productivity of BC formation from mannitol varies over a fairly wide range and depends on the specific characteristics of the producing culture, in many cases exceeding the levels achieved on mostly used carbon sources (glucose or fructose). Thus, when cultivated under static conditions with 1.5% mannitol as the sole carbon source, *A. xylinum* KU-1 achieved relatively high BC concentration and specific productivity of 4.6 g/L and 0.027 g/L/h, respectively. Under the same conditions, these indices were significantly lower (1.2 g/L and 0.007 g/L/h) for 1.5% glucose (Oikawa et al. 1995a). In addition, no gluconic acid accumulates in the mannitol medium, thus maintaining a pH value about 6.0 favorable for BC synthesis, while unwanted acidification (pH 3.4) is observed on the glucose (Oikawa et al. 1995a). Moreover in another study with *A. xylinum sp*. it was shown that the use of mannitol (50 g/L) in a longer-time cultivation under static conditions makes it possible to achieve a relatively high BC yield (about 4.75 g/L), but with lower specific productivity (0.014 g/L/h), which still exceeds the values for glucose as the carbon source, but are commensurate with or slightly below the indices for sucrose medium also depending on the nitrogen sources used (Ramana et al. 2000). In addition, when growing *Gluconacetobacter hansenii* ATCC 10821 on 2% mannitol the BC yield and productivity were 20–25% above that in the glucose medium but remained rather low (1.13 ± 014 g/L; 0.0021 g/L/h) under selected (20 °C; pH 5.5) static culture conditions (Hutchens et al. 2007). Similar data on the use of mannitol for BC synthesis with a higher productivity as compared to glucose, fructose or sucrose were also obtained with other producer cultures such as *G. xylinus* ATCC10245 (El-Saied et al. 2008), *G. xylinus* K3 (Nguen et al. 2008), wild-type isolates *G. xylinus sp*. S and *G. xylinus sp*. A2 (Jalili Tabaii and Emtiazi 2016), *Gluconacetobacter sp.* isolate PAP1 (Suwanposri et al. 2013), as well as *G. xylinus* ATCC 53524 (Mikkelsen et al. 2009), but in this case only for short (48 h) cultivation and later (96 h) slightly lagging behind the productivity of sucrose. A somewhat higher BC yield and specific productivity (3.9 g/L; 0.163 g/L/h) in the 24-h cultivation in shaking flasks (140 rpm) on mannitol as compared to glucose (3.2 g/L; 0.133 g/L/h) were also confirmed for the producer strain *A. aceti* MTCC2623 (Dayal et al. 2013). However, differing effects of mannitol on BC yield and productivity have also been reported (Son et al. 2001; Jung et al. 2010). Thus, when *Acetobacter sp.* A9 was used as a producer (Son et al. 2001), the yield of BC on 2% mannitol (0.64 g/L) proved to be substantially below that for glucose (2.70 g/L), fructose (2.53 g/L) and sucrose (0.83 g/L), as well as specific productivity (0.013 g/L/h, 0.056 g/L/h, 0.053 g/L/h and 0.017 g/L/h, respectively). Similar characteristics were also observed for *Acetobacter sp.* V6 (Jung et al. 2010) when only 0.45 g/L BC was obtained in the 7-day cultivation on mannitol, significantly lagging behind glucose (1.13 g/L), although well above the yield for sucrose (0.14 g/L). Substantially higher yields of BC from glucose, fructose, and sucrose compared to mannitol were observed using the producer strain *G. xylinus* PTTC 1734 (Jalili Tabaii and Emtiazi 2016), as well as from fructose in the case of *Komagataeibacter sp*.W1 (Wang et al. 2018) or from glucose using *G. sacchari sp*. isolated from Kombucha tea (Trovatti et al. 2011). Some recent studies on the use of mannitol in the production of BC have shown fairly good yield and productivity characteristics using the producers *K. xylinus* SB3.1 (Alemam et al. 2021) and *K. xylinus* K2G30 (UMCC 2756) (Gullo et al. 2019). Their levels, especially in the case of K2G30 (8.77 g/L; 0.041 g/L/h), indicate some potential for the industrial scale-up of BC production using vegetable waste feedstocks rich in mannitol and other sugar alcohols (Gullo et al. 2019). In addition, during cultivation these carbon sources prevent the formation of gluconic acid, which decrease the pH far below the optimum value for BC production (Oikawa et al. 1995a; Gullo et al. 2019). Compared to mannitol, other sugar alcohols, such as arabitol and xylitol were used much less frequently as carbon sources for BC synthesis, and the yields and productivity obtained were rather low or moderate (Son et al. 2001; Singhsa et al. 2018; Gullo et al. 2019).

**Fig. 1:**
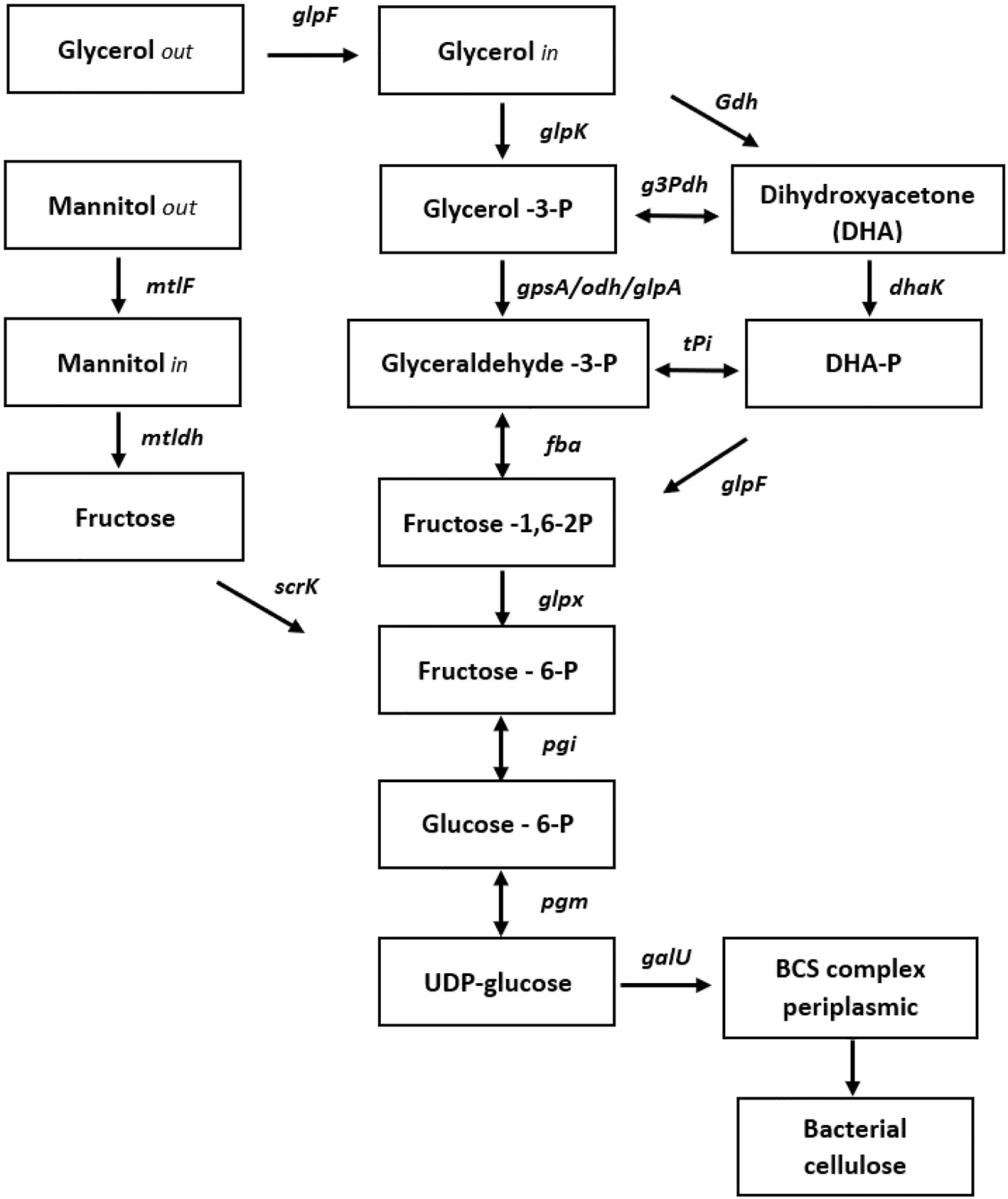
Metabolic pathways of bacterial cellulose synthesis from glycerol and mannitol by acetic acid bacteria. Gene names and gene products. Adapted from Azuma et al. 2009 and Lu et al. 2020. Gene names and gene products: pgi: glucose-6-phosphate isomerase; glpX: fructose-1,6-bisphosphatase II; fba: fructose-bisphosphate aldolase, class II; pgm: phosphoglucomutase; galU: UTP-glucose-1-phosphate uridylyl transferase; glpK: glycerol kinase; gpsA/odh/glpA: glycerol-3-phosphate dehydrogenase; glpF: glycerol facilitator; dhaK: dihydroacetone kinase; gdh: glycerol dehydrogenase; g3pdh: glyceraldehyde-3-phosphate dehydrogenase; mtlF: mannitol facilitator; mtldh: mannitol dehydrogenase

The fact that generally the yield and productivity of BC on arabitol and xylitol remain below that on mannitol is due to the differences in respective metabolic pathways. Thus, a soluble and highly substrate-specific D-mannitol dehydrogenase (Oikawa et al. 1997) realizes a direct enzymatic conversion of mannitol to fructose, which is subsequently phosphorylated to fructose-6-P to be involved in the further metabolic pathway to BC (Fig. 1). Contrary to D-mannitol, the production of BC from arabitol or xylitol is a more complex pathway, involving additional steps, such as the intermediate formation and phosphorylation of xylulose, followed by conversion to glyceraldehyde-3-P to enter the main stream (Fig. 1) of biosynthesis (Bettiga et al. 2008; Laslo et al. 2012; Glenn et al. 2014), whose result is less advantageous energetically for the cell (Gullo et al. 2019). Although using the producer *A. xylinum* KU-1, a very high BC yield and productivity (12.4 g/L; 0.129 g/L/h) were achieved on arabitol, which is higher than that for mannitol, and even six times more (2.0 g/L; 0.021 g/L/h) than for glucose (Oikawa et al. 1995a, b; Oikawa et al. 1997), also suggesting the possibility of activation for this pathway under certain conditions and, consequently, the full use of 5-C sugar alcohols from vegetable raw materials (Laslo et al. 2012; Gullo et al. 2019).

## Glycerol as an appropriate feedstock for the synthesis of bacterial cellulose

However, despite such manifestations of individual polyols, it is glycerol that has a clear advantage for the use as a raw material in the production of bacterial cellulose. Thus, in view of the large volume of glycerol that accompanies the ever-increasing production of diesel fuel from renewable fats and oils (da Silva et al. 2009; Wendisch et al. 2011; Trindade et al. 2015), almost all restrictions on the availability and prices of carbon sources for BC biosynthesis have been removed (Vazquez et al. 2013). In addition, glycerol easily enters the metabolic network of producing cells (da Silva et al. 2009), by facilitated diffusion (Fig. 1), while other also renewable and cost-efficient feedstocks, such as molasses or cheese whey, require for this an enzymatic or chemical hydrolysis of relevant disaccharides (sucrose and lactose, respectively). This need is due to the fact that, for example, the activity of β-galactosidase in AAB is often too small for efficient hydrolysis of lactose to obtain glucose for BC synthesis (Lappa et al. 2019; Zikmanis et al. 2020a, b). In turn, sucrose cannot be transported across the cell membrane and is hydrolyzed in the periplasm to glucose and fructose by α-glucosidase, which has very varied and often insufficient activities for different producer strains (Velasco-Bedrán and López-Isunza 2007; Mikkelsen et al. 2009; Jalili Tabaii and Emtiazi 2016; Raghavendran et al. 2020). Therefore, these substrates should be subjected to preliminary hydrolytic treatment using appropriate enzymes or less expensive chemical hydrolysis with mineral acids (Marangoni et al. 2002; Bae and Shoda 2004; Torres et al. 2010; Kucera et al. 2018). The efficiency of using glycerol is also determined by the fact that the necessary enzymatic stages of its catabolism are well-represented in the metabolic network of BC producers (Azuma et al. 2009; Lu et al. 2020). For this, alternative ways are possible (Fig. 1), such as inclusion in the biosynthesis chain at the level of glyceraldehyde-3-P after phosphorylation of glycerol with glycerol kinase (glpK) and subsequent transformation by glycerol-3-P dehydrogenase (gpsA/odh/glpA) or through the intermediate formation of dihydroacetone (DHA) by glycerol dehydrogenase (Gdh) phosphorylation to DHA-P with dihydroacetone kinase (dhaK) and isomerization by triose-P-isomerase (tPi), DHA-P can also enter at the fructose-1.6-2P level (Fig. 1) after transformation by fructose-biphosphate aldolase (fba). Further enzymatic transformations, as for all other substrates, pass through the formation of glucose-6-phosphate, which is converted to glucose-1-phosphate and subsequently metabolized to uridine diphosphoglucose (UDP-glucose), a direct precursor of BC. UDP biosynthesis is controlled by phosphoglucomutase (pgm) and UTPG-1-P uridylyl transferase (galU) also referred as UDPG pyrophosphorylase (Thoden and Holden 2007; Lynch et al. 2019). After the formation of UDP-glucose, the polymerization of glucose into BC is mediated by cellulose synthase (CS), a complex of proteins spanning the periplasmic space between the bacterial cytoplasmic and outer membranes. The CS complex promotes UDP-glucose polymerization, polymer translocation across bacterial membranes, and extracellular assembly of glucan chains (Tonouchi 2016; Lynch et al. 2019). The activities and structure of CS in detail have been discussed in several reviews (Adnan 2015; McNamara et al. 2015; Jang et al. 2017; Gullo et al. 2018; Raghavendran et al. 2020). It is important to note that the CS complex is encoded by a four-gene (bcsA, bcsB, bcsC, and bcsD) operon which determines the formation and activity of the corresponding elements, including the catalytic (AcsA) and regulatory (BcsB) subunits. In turn, their activity and, therefore, the entire complex is under allosteric regulatory control by means of the cyclic diguanosine monophosphate (c-di-GMP), the binding of which to the BcsB regulatory subunit activates the AcsA catalytic subunit via its C-terminal transmembrane helix (Lu et al. 2020; Buldum et al. 2018; Römling and Galperin 2015). The degree of activation depends on the level of cellular c-di-GMP, which is determined by several external and internal factors such as oxygen availability, intracellular potassium concentration, diguanylate cyclase and phosphodiesterase activities (Gullo et al. 2018; Raghavendran et al. 2020) Assessing the above problems as a whole, it becomes quite clear why the studies on the use of glycerol for the production of BC, although not very numerous, significantly exceed the current volume of relevant studies using molasses (Adnan 2015; Campano et al. 2016; Zhong 2020) or cheese whey (Kolesovs and Semjonovs 2020; Zikmanis et al. 2020a, b). The main results on the use of glycerol for BC synthesis are summarized in Table 1.

**Table 1:** Production of bacterial cellulose by acetic acid bacteria from pure or crude glycerol as the sole carbon source

Bacterial strain	Growth medium	Cultivation mode	Medium composition	Production metrics^a^	References
*Komagataeibacter sucrofermentans* DSM15973	HS	Static	20 g/L pGLYC or 20 g/L cGLYC, 5 g/L YE, 5 g/L Pep, 1.1 g/L citric acid	pGLYC 1.9 g/L (0.008 g/L/h) cGLYC 6.4 g/L (0.027 g/L/h)	Lee et.al. (2021)
*K. hansenii* ATCC 53582	HS	Static	2% pGLYC, 0.5% YE, 0.5% Pep	4.93 g/L (0.068 g/L/h)	Li et al. (2021)
*K. rhaeticus* ENS9a	MA/9	Static	20 g/L pGLYC 20 g/L cGLYC	2.6 g/L (0.01 g/L/h) 2.9 g/L (0.01 g/L/h)	Mangayil et al. (2021)
*A.senegalensis* MA1	HS	Static	63 g/L pGLYC, 7.5 g/L YE; 7.76 g/L PEG 6000	469.83 g/L (0.652 g/L/h)^**b**^	Aswini et al. (2020)
*G. xylinus* sp.	HS	Static	20 g/L cGLYC, 5 g/L YE, 5 g/L Pep 40 g/L cGLYC, 5 g/L YE, 5 g/L Pep	1.5 g/L(0.01 g/L/h) 2.9 g/L (0.02 g/L/h)	Dikshit and Kim (2020)
*Komagataeibacter* sp.nov CGMCC 17,276	AE	Static	20 g/L pGLYC, 9 g/L YE, 5 g/L Pep, 30 g/L CSL, 0.3% AA, 1.5% Eth; 40 g/L cGLYC 5 g/L YE, 5 g/L Pep 30 g/L CSL, 0.7% AA, 0.3% Eth	4.5 g/L (0.05 g/L/h) 6 g/L (0.06 g/L/h)	Lu et al. (2020)
*K. hansenii* JR-02	HS modified	Static	20 g/L pGLYC, 2.5 g/L YE, 2.5 g/L Pep	2.4 g/L (0.01 g/L/h)	Li et al. (2019)
*G. xylinus* ATCC 23769	HS	Static	30 g/L cGLYC, 16 g/L YE, 4 g/L Na_2_HPO_4_, 3.5 g/L succinic acid	0.24 g/L (1.4 mg/L/h)	Lins et al. (2019)
*A. xylinum* sp.	HS	Static	40 g/L cGLYC, 5 g/L YE, 5 g/L Pep	1.5 g/L (4 mg/L/h)	Wu et al. (2019)
*G. xylinus* KCCM 41431	HS	Static	20 g/L cGLYC, 9 g/L YE, 9 g/L Pep 20 g/L pGLYC, 9 g/L YE, 9 g/L Pep	7.4 g/L (0.041 g/L/h) 7.7 g/L (0.044 g/L/h)	Yang et al. (2019)
*Komagataeibacter saccharivorans* sp.	HS	Static	20 g/L cGLYC, 5 g/L YE, 5 g/L Pep	12.6 g/L (0.08 g/L/h)	Gayathri and Srinikethan (2018)
*G. xylinus* BNKC19	HS	Static	10 g/L cGLYC, 5 g/L YE, 5 g/L Pep	12.3 g/L (0.07 g/L/h)	Soemphol et al. (2018)
*K. rhaeticus* PG2	HS	Static	20 g/L pGLYC, 5 g/L YE, 5 g/L Pep 30 g/L pGLYC, 5 g/L YE, 5 g/L Pep	6.9 g/L (0.02 g/L/h) 8.7 g/L (0.02 g/L/h	Thorat and Dastager (2018)
*K. xylinus* B-12068	HS modified	Static	20 g/L pGLYC, 5 g/L YE, 5 g/L Pep, 3% Eth	23.2 g/L (0.14 g/L/h)	Volova et al. (2018)
*Komagataeibacter* sp. W1	HS	Static	20 g/L pGLYC, 5 g/L YE, 5 g/L Pep	1.2 g/L (4 mg/L/h)	Wang et al. (2018)
*Gluconoacetobacter xylinus* DSM46604	Defined, YE containing	Shake flasks agitated Bioreactor 3L, agitated/aerated	20 g /L pGLYC, 5 g/L YE, 5 g/L (NH_4_)_2_SO_4,_ 3 g/L K_2_HPO_4_, 0.05 g/L MgSO_4_	1.43 g/L (0.012 g/L/h) 2.87 g/L (0.024 g/L/h)	Adnan et al. (2015) Adnan (2015)
*Gluconoacetobacter* sp. A2	HS	Static	20 g/L pGLYC,5 g/L YE, 5 g/L Pep	1.95 g/L (0.027 g/L/h)	Jalili Tabaii and Emtiazi (2016)
*K. sucrofermentans* DSM 15,973	HS	Static	20 g/L cGLYC,5 g/L YE, 5 g/L Pep	3.2 g/L (0.01 g/L/h)	Tsouko et al. (2015)
*Acetobacter xylinum* AJ_3_	Defined,YE,peptone containing	Static	35 g/L pGLYC,10 g/L YE, 7.5 g/L Pep,10 g/L Na_2_HPO_4_, 10 g/L AA	8.52 g/L (0.044 g/L/h)	Al-Shamary and Al-Darwash (2013)
*A. xylinum* DSMZ-2004	Semidefined, YE, apple extract containing	Static	2% pGLYC, 2.5% glucose (apples),0.05% YE, 0.3% (NH_4_)_2_SO_4_, 0.5% citric acid	8.6 g/L (0.026 g/L/h	Casarica et al. (2013)
*G. intermedius* NEDO-01	HS	Shake flasks rotating Bioreactor 5L, agitated/aerated	20 g/L cGLYC,5 g/L YE, 5 g/L Pep, 25 g/L CMC	1.3 g/L (0.01 g/L/h) 3.4 g/L (0.034 g/L/h)	Kose et al. (2013)
*G*. *sucrofermentans* CECT 7291	HS	Static	20 g/L pGLYC, 5 g/L YE, 5 g/L Pep, 2.7 g/L Na_2_HPO_4_, 1.15 g /L citric acid	2.0 g/L (0.0064 g/L/h)	Santos et al. (2013)
*G. xylinus* CGMCC 2955	Defined	Shake flasks agitated	25 g/L pGlyc, 3 g/L Na2HPO4, 1 g/L KH2PO4, 5 g/L (NH4)2SO4, 0.02 g/L MgCl2, 0.02 g/LCaCl2, 0.0015 g/L paraaminobenzoic acid	6.05 g/L (0.050 g/L/h)	Zhong et al. (2013)
*G. sacchari* sp.	HS	Static	20 g/L cGLYC, 5 g/L YE, 5 g/L Pep	0.1 g/L (0.1 mg/L/h)	Carreira et al. (2011)
*G. persimmonis* GH-2	HS	Bioreactor 5L, agitated/aerated	20 g/L pGLYC,5 g/L YE, 5 g/L Pep	2.47 g/L (0.013 g/L/h)	Hungund and Gupta (2010a)
*Enterobacter amnigenus* GH-1	HS	Static	20 g/L pGLYC,5 g/L YE, 5 g/L Pep	1.2 g/L (0.004 g/L/h)	Hungund and Gupta (2010b)
*Acetobacter* sp. V6	HS modified	Shake flasks agitated	2% pGLYC, 1.6% YE, 0.4% Na_2_HPO4, 0.35% succinic acid	4.98 g/L (0.030 g/L/h)	Jung et al. (2010)
*G. xylinus* ATCC 53,524	HS	Static	20 g/L pGLYC, 5 g/L YE, 5 g/L Pep, 2.7 g/L Na_2_HPO_4_, 1.15 g /L citric acid	3.75 g/L (0.039 g/L/h)	Mikkelsen et al. (2009)
*Gluconacetobacter* sp. RKY5	HS optimized	Static Shake flasks agitated	15 g/L pGLYC, 8 g/L YE, 3 g/L K_2_HPO_4_, 3 g/L AA	4.59 g/L (0.032 g/L/h) 5.63 g/L (0.039 g/L/h)	Kim et al. (2006)
*G. xylinus* ATCC 10,245	HS	Static	10 g/L pGLYC,5 g/L YE, 5 g/L Pep, 2.7 g/L Na_2_HPO_4_, 1.15 g/L citric acid		Keshk and Shameshima (2005)
*A. xylinum* DA	YPD	Static	20 g/L pGLYC, 5 g/L YE, 5 g/L Pep, 2.7 g/L Na_**2**_HPO_4_ , 1.15 g /L citric acid, 20 g/L AA	3.83 g/L (0.016 g/L/h)	Toda et al. (1997)
*A.xylinum* IFO 13,693	HS modified	Shake flasks agitated	0.5% pGLYC,0.5% YE, 2% Pep,0.1% MgSO_4_,0.2% Eth	4.84 g/L (0.067 g/L/h)	Masaoka et al. (1993)

*CMC* carboxymethyl cellulose, *HS* Hestrin–Schramm medium, *YPD* yeast extract–peptone–glucose medium, *AE* acetate ethanol medium, *MA/9* unoptimized minimal medium, *pGLYC* pure glycerol, *cGLYC* crude glycerol, *YE* yeast extract, *Pep* peptone, *Eth* ethanol, *AA* acetic acid, *CSL* corn steep liquor

^a^BC concentration at the end of cultivation (specific productivity: increase in concentration on average per hour)

^b^Both expressed in wet weight

Producer cultures, whose ability to synthesize bacterial cellulose from glycerol have been documented, represent exclusively acetic acid bacteria (Table 1) of the genus *Komagataeibacter* (formerly *Acetobacter*, *Gluconacetobacter*) with only one exception for conditionally pathogenic *Enterobacter amnigenus* (Hungund and Gupta 2010b). Although these studies were mainly performed with organisms of the same species (*K. xylinus* or *K. rhaeticus*), in all cases different strains of producers were used, both from representative collections (ATCC, DSMZ, NRRL, etc.) of microorganisms and from relatively less characterized isolates of different origins. This characterizes both the widespread ability of AAB to biosynthesize BC from glycerol and makes it difficult to compare the data to select the most promising producer based on its productivity. It can be seen (Table 1) that relevant indicators such as the yield and specific productivity vary over a very wide range, from the relatively insignificant (Carreira et al. 2011; Lins et al. 2019) to quite high levels, confirming the biotechnological potential of such producers (Vazquez et al. 2013; Volova et al. 2018; Soemphol et al. 2018; Vigentini et al. 2019). These data are in line with the oft-confirmed finding that microbial synthesis of extracellular polysaccharides is highly strain-dependent (Olivas and Barbos-Canovas 2005; Nwodo et al. 2012; Devi and Alamu 2013; Semjonovs et al. 2017b), including the formation of BC from glycerol, and thus suggest that further in-depth comparative studies of relevant genotypic and phenotypic traits of potential producers are urgently needed. This is especially evident in the fact that bacterial sub-clones with persistent phenotypic properties and markedly different productivity of BC synthesis can be isolated even from a supposedly homogeneous producer strain, such as *K. rhaeticus* LMG 22126 (Vigentini et al. 2019). Besides, the composition of the cultivation medium and the chosen operational conditions also are very important to promote the BC biosynthesis. Producer cultures were predominantly cultivated in a standard (Mikkelsen et al. 2009; Tsouko et al. 2015; Thorat and Dastager 2018; Soemphol et al. 2018) or modified (Jung et al. 2010; Volova et al. 2018; Li et al. 2019) Hestrin–Schramm (HS) medium commonly used for acetic acid bacteria, replacing conventional carbon sources (mono- or di-saccharides) with glycerol. In some cases, other nutrient media of defined (Al-Shamary and Al-Darwash 2013; Lu et al. 2020; Vigentini et al. 2019; Mangayil et al. 2021), semi-defined (Casarica et al. 2013) or complex (Vazquez et al. 2013) compositions were also employed. In a number of studies, a positive effect of increased glycerol concentrations on cellulose yield and specific productivity of the bioprocess as a whole was noted. Besides, the addition of various additives such as organic acids (Toda et al. 1997; Kim et al. 2006; Jung et al. 2010; Lins et al. 2019), ethanol (Masaoka et al. 1993; Volova et al. 2018; Lu et al. 2020), water-soluble polymers (Kose et al. 2013) to the culture medium can also significantly promote the formation of BC. By this time, purified glycerol is predominantly included in the composition of nutrient media, although a fairly high productivity for BC is also achieved when this source is used in a crude, i.e., not purified, form (Vazquez al. 2013; Gayathri and Srinikethan 2018; Yang et al. 2019; Lu et al. 2020). Quite comparable levels of BC yields are also shown for some producers when using both types of glycerol, which can be facilitated by the more appropriate composition of nitrogen sources (Lu et al. 2020). It should be noted that nutrient formulations currently use almost exclusively traditional nitrogen sources, such as yeast extract (YE) and/or peptone (Pep), although good potential has also been shown (Vazquez et al. 2013) for corn steep liquor (CSL). Replacing traditional nitrogen sources not only with the less expensive CSL, but also with other nitrogen-rich extracts, concentrates and hydrolysates of natural origin is crucial for any further scaling of BC biosynthesis processes, as this would significantly reduce their overall costs. Such attempts using fish powder, soybean meal, malt extract, casein or fish hydrolysates in combination with glycerol have been performed with some success (Hungund and Gupta 2010b; Adnan 2015; Aswini et al. 2020). However, further in-depth studies of their optimal levels are required to maximize BC formation for different producers, at least commensurate with the productivity achieved by traditional sources. The situation is quite similar to the use of crude glycerol for the production of BC. It is well known that not purified glycerol contains a number of by-products (methanol, soap, inorganic salts, free fatty acid, unreacted acyl glycerols, etc.) whose composition and amounts are affected by possible differences in the biodiesel technologies used (Samul et al. 2013; Mangayil et al. 2021). Although crude glycerol (Wendisch et al. 2011; Posada et al. 2012), like purified glycerol (Kenar 2007; da Silva et al. 2009), is successfully used to obtain many value-added products, including BC, by microbial conversion, such impurities can also adversely affect the activity of producer cells and the overall biosynthetic pathway. Such effects can be eliminated by some pre-treatment of crude glycerol, for which various partial or more complete purification methods have been developed (Posada et al. 2012; Yang et al. 2012). However, this can significantly increase the overall cost and should therefore be avoided. Thus, in order to ensure a high yield of BC, which is undoubtedly achievable on a crude glycerol (Vazquez al. 2013; Gayathri and Srinikethan 2018; Yang et al. 2019; Lu et al. 2020), it is necessary to balance the physiological requirements of different producers for the substrate qualities with its composition at different origins through appropriate focused research for the accumulation and comparative analysis of more data (Samul et al. 2013). This would provide still absent, more general recommendations for the use of crude glycerol, thereby contributing to the cost efficiency of BC production. Compared with the mode of static cultivation (Table 1), BC production under aeration/agitation conditions has been studied much less frequently (Kim et al. 2006; Jung et al. 2010; Kose et al. 2013; Adnan et al. 2015), especially when using bioreactors (Hungund and Gupta 2010b; Adnan 2015). The yield and productivity achieved by BC are also quite moderate (Kim et al. 2006) not exceeding 5.63 g/L (0.039 g/L/h), thus lower compared to the several static cultivation data (Table 1). However, further research is needed in this direction, as it is well known that higher oxygen supply and volumetric agitation could promote increasing yields and specific productivity for producer strains, thus amplifying the bioprocess towards the large-scale industrial production

## Conclusions

Glycerol as an undesirable and polluting by-product, i.e., waste generated in significant quantities in combination with biodiesel production requires greater re-use, including the production of valuable products through microbiological conversion.

This polyol, even in a crude unrefined form, can be used as a renewable carbon source for the production of BC, which could stimulate the development of cost-efficient and environmentally friendly technologies to achieve an expanded use of this biopolymer within a broad and versatile range of practical applications. Since the data obtained so far remain somewhat one-sided and largely limited, the relevant research needs further development.

As with other carbon sources, the microbial synthesis of BC from glycerol is highly strain-dependent, that is, the yield, specific productivity and structural features of a biopolymer are determined by specific properties, both genotypic and phenotypic, of the producer. In turn, this creates the need for thorough studies and assessment of these properties, even for seemingly homogeneous strains for their appropriate full-fledged selection.

A much wider use of appropriate optimization techniques is required to identify and determine the optimal levels of the relevant factors for maximum BC formation by selected producers.

The inclusion of various nitrogen-rich extracts and hydrolysates of natural origin, which is still a rather rare, is particularly important in the development and optimization of cultivation media compositions, as this would significantly reduce the overall cost of BC synthesis.

There is a need to significantly expand the research on BC production from glycerol under aerated/agitated conditions, which are still noticeably less used than static cultivation, although they could promote increasing yields and specific productivity. However, the effect of such conditions should be additionally assessed in terms of their potential impact on the structural properties of BC and, consequently, on the suitability of the polymer for specific applications.

## Data Availability

The datasets used and/or analyzed during the current study are available from the first author on reasonable request and/or are freely accessible via the Internet.
